# Complete Genome Sequence of Bacillus licheniformis Strain GN02, Isolated from the Root Surface of Brassica chinensis

**DOI:** 10.1128/mra.00092-23

**Published:** 2023-05-03

**Authors:** Yan He, Jinzhi Xu, Lijun Qin, Xingyong Yang

**Affiliations:** a College of Life Science, Chongqing Normal University, Chongqing, China; b College of Pharmacy, Chengdu University, Chengdu, China; University of Arizona

## Abstract

Bacillus licheniformis GN02 was isolated from the root surface of Pak Choi Cabbage (Brassica chinensis). Here, we report the whole-genome sequence of strain GN02, which includes only a circular chromosome (4,252,022 bp; GC content, 46.08%).

## ANNOUNCEMENT

Bacillus licheniformis is a Gram-positive, spore-forming, facultatively anaerobic bacterium, closely related to Bacillus subtilis in phylogeny. It is applied to all aspects of agriculture, food, and the biotechnology industry, for example, as a biocontrol agent, antibiotic enzyme, and biochemical (1, 2). According to the method of Shen et al. (3), N_2_-fixing Ashby agar medium (28°C for 3 to 5 days) was used in an isolation assay. On 12 August 2013, B. licheniformis GN02 was isolated from the root surface of Pak Choi Cabbage (Brassica chinensis L.) in Beibei District (30°26′12″N, 106°26′25″E; Chongqing, China). Here, we sequenced the complete genome of strain GN02 to further clarify its plant growth-promoting mechanisms.

Isolate GN02 was cultured in nutrient broth by shaking (120 rpm) overnight at 28°C, and its 16S rRNA sequence was amplified by colony PCR using universal primers (27F/1492R). GN02 was identified as B. licheniformis based on its closest match (identity, 100%; E value, 0), B. licheniformis P8_B2 (GenBank accession number CP045814.1), using the online NCBI BLAST tool (standard databases, nonredundant/nucleotide [nr/nt] collection [accessed 12 January 2023]; optimized for “Highly similar sequences-megblast”). DNA was extracted from enriched bacterial cells and sequenced by Guangzhou Genedenovo Biotechnology Co., China, using the RS II system (Pacific Biosciences, CA). Based on the manufacturer’s recommendations, 20-kbp insert whole-genome shotgun sequencing libraries were prepared using 0.45× volumes of Agencourt AMPure XP beads (Beckman Coulter Genomics, MA). To evaluate the complexity of the genome and correct the PacBio long reads, the genome of GN02 was sequenced by Shanghai Biozeron Co., China, using the HiSeq platform (paired-end, 2 × 150-bp [PE150] mode; Illumina, USA). Paired-end libraries with insert sizes of ~400 bp were prepared using TruSeq DNA library prep kits (Illumina) following the standard procedure. Finally, 9,999,176 raw paired-end Illumina reads (average length, 149 bp) and 90,806 PacBio reads (average length, 10,149.16 bp; *N*_50_, 14,107 bp) were generated. The Illumina data were quality filtered using Trimmomatic v0.32 (4), and the PacBio data were assembled into a contig using Unicycler v0.4.8 (5). GapCloser v1.12 (6) was subsequently applied to fill the remaining local inner gaps and correct the single base polymorphism for the final assembly results. An assembly contig of 4,252,022 bp was finalized as the circular chromosome, with a GC content of 46.08%.

Using RNAmmer v1.2 (7) to predict rRNAs, 8 copies each of 5S rRNAs, 16S rRNAs, and 23S rRNAs were predicted. Using tRNAscan-SE v2.0 (8), 81 copies of tRNAs for 20 types of tRNAs were predicted. Further, 11 gene islands were predicted using IslandPath-DIMOB v1.0.0 (9). There were 4,302 coding genes (CDS) predicted using Glimmer v3.02 (10). Unless otherwise noted, default parameters were used for all software.

### Data availability.

The project data for Bacillus licheniformis strain GN02 have been submitted under GenBank accession number CP116941. The raw data have been deposited in the SRA under accession numbers SRR22354641 (PacBio) and SRR22354640 (Illumina). The BioSample and BioProject accession numbers are SAMN31807140 and PRJNA903518, respectively.
